# In vivo synergistic enhancement of MIF‐mediated inflammation in acute lung injury by the plant ortholog *Arabidopsis* MDL1

**DOI:** 10.1096/fj.202403301R

**Published:** 2025-03-26

**Authors:** Lukas Spiller, Lin Zhang, Simona Gerra, Christian Stoppe, Patrick Scheiermann, Thierry Calandra, Elias Lolis, Ralph Panstruga, Jürgen Bernhagen, Adrian Hoffmann

**Affiliations:** ^1^ Division of Vascular Biology, Institute for Stroke and Dementia Research (ISD) LMU University Hospital, Ludwig‐Maximilians‐Universität (LMU) Munich Munich Germany; ^2^ Department of Pharmacology, Yale School of Medicine Yale University New Haven Connecticut USA; ^3^ Department of Cardiac Anaesthesiology and Intensive Care Medicine Charité Berlin Germany; ^4^ Department of Anaesthesiology, Intensive Care, Emergency and Pain Medicine University Hospital Würzburg Würzburg Germany; ^5^ Department of Anaesthesiology LMU University Hospital, Ludwig‐Maximilians‐Universität (LMU) Munich Munich Germany; ^6^ Service of Immunology and Allergy, Department of Medicine and Department of Laboratory Medicine and Pathology, Center for Human Immunology Lausanne University Hospital, University of Lausanne Lausanne Switzerland; ^7^ Center for Advanced Studies Ludwig‐Maximilians‐Universität (LMU) Munich Munich Germany; ^8^ Unit of Plant Molecular Cell Biology, Institute for Biology I RWTH Aachen University Aachen Germany; ^9^ German Centre of Cardiovascular Research (DZHK), Partner Site Munich Heart Alliance Munich Germany; ^10^ Present address: Boehringer Ingelheim Pharma GmbH & Co. KG, Global Clinical Development & Operations Ingelheim am Rhein Germany

**Keywords:** acute lung injury, atypical chemokine, MDL, MIF, plant MIF

## Abstract

Recent research has uncovered *Arabidopsis thaliana* proteins that are similar to the human inflammatory cytokine MIF. Plant MIF/D‐dopachrome tautomerase (D‐DT)‐like proteins (MDLs) can interact with human MIF, yet the significance of these findings in living organisms has not been investigated. Given MIF's key role in acute respiratory distress syndrome promoting pulmonary inflammation, pathology, and leukocyte infiltration, here we set out to investigate the interplay between MIF and MDL1, one of three *A. thaliana* MIF orthologs, in an in vivo mouse model of MIF‐induced acute lung injury (ALI). Human MIF and MDL1 were administered to C57BL/6 mice via inhalation, individually or in combination. Inhalation of MIF promoted various parameters of lung injury as evaluated by flow cytometry, immunofluorescence microscopy, RT‐qPCR, and ELISA, while MDL1 inhalation alone had no effect. Intriguingly, combined treatment with MIF and MDL1 synergistically enhanced pulmonary infiltration of neutrophils and monocytic cells, accompanied by an upregulation of pro‐inflammatory cytokine genes. Thus, the plant‐derived MIF ortholog MDL1 potentiates MIF‐induced inflammation in ALI. These data support the growing evidence of interactions between plant‐derived compounds and human inflammatory mediators and illustrate how they may impact human health.

## Graphical abstract

Scheme of the synergistic enhancement of MDL1 on MIF‐induced parameters of pulmonary inflammation. Although without measurable effect when administered alone, plant MDL1 amplifies MIF‐induced expression of key inflammatory mediators and leukocyte recruitment most likely via complex formation.

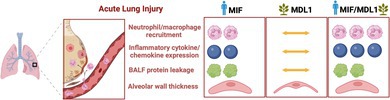

## 1 INTRODUCTION

Interactions between the human immune system and pathogens such as bacteria, viruses, or parasites are well defined. Plants also interact with components of the host defense machinery, for example after contact with skin, inhalation of plant particles, or upon ingestion. However, aside from plant‐derived antigens (causing allergies) and medicinal compounds, human–plant immune interactions remain poorly characterized.

As part of an effort to characterize cross‐kingdom interactions affecting the human immune system, we recently identified orthologs of the human cytokines macrophage migration inhibitory factor (MIF) and D‐dopachrome tautomerase (D‐DT, *aka* MIF‐2) in the reference plant *Arabidopsis thaliana* and other plant species.^1^, ^2^, ^3^ These proteins have been named MIF/D‐DT–like (MDL) proteins. MIF is a multifunctional chemokine‐like inflammatory cytokine and has been associated with a wide range of acute and chronic immune and inflammatory diseases as well as cancer.^1^, ^4^, ^5^ MIF signaling and target‐cell activities are mediated by interactions with its cognate receptor CD74 and the chemokine receptors CXCR2, CXCR4, and ACKR3.^4^, ^6^ The widespread presence of MDL proteins across kingdoms suggests that MIF may have evolved as an ancient enzyme and later on adopted functions as an extracellular‐acting cytokine/chemokine.^3^

Recently, we resolved the X‐ray structures of all three *A. thaliana* MDL proteins (MDL1‐3), revealing high 3D‐structural similarity to MIF, while uncovering differences in the tautomerase catalytic cavity and receptor‐interacting determinants.^2^ Of note, biochemical and functional in vitro assays indicated that MDLs can form hetero‐oligomeric complexes with MIF that trigger synergistic signaling.^2^ These observations call for studies in in vivo models to explore the pathophysiological relevance of MDL‐MIF interactions. Given MIF's established disease‐aggravating roles in acute respiratory distress syndrome (ARDS), we here aimed to study potential immunomodulatory cross‐kingdom interactions between MIF and MDL1 in an in vivo mouse model of ALI, which reflects the acute and sub‐acute phases of ARDS.^5^, ^7^

## 2 MATERIALS AND METHODS

### 2.1 Protein Expression

Recombinant human MIF and *A. thaliana* MDL1 proteins were produced using a bacterial overexpression system and purified by ion metal‐affinity chromatography (His‐Trap) and size‐exclusion chromatography (Superdex 75 10/300) as described.^2^, ^4^ For details see Supplementary Methods. Mouse MIF was produced as reported.^8^

### 2.2 In vivo‐mouse model of ALI

Healthy male C57BL/6 mice were used to assess the effects of MIF, MDL1, and their combination on lung inflammation. Mice (10–12 weeks old) were exposed to proteins (500 μg/mL), saline, or lipopolysaccharide (LPS, *Salmonella enteritidis*, Sigma‐Aldrich) (500 μg/mL) via nebulizer for 30 min as established.^9^ After 12 h, the mice were sacrificed, and bronchoalveolar lavage fluid (BALF), lung tissue, and blood were collected. All procedures were approved by the local animal care committee (Regierung von Oberbayern, ROB‐55.2_Vet_2532.Vet_02–18–40).

### 2.3 Flow cytometry

Flow cytometry of mouse blood and enzymatically digested lung tissue single cell suspensions (LiberaseTM, Sigma‐Aldrich) was used to assess neutrophil and monocytic cell counts. Cells were stained with fluorochrome‐conjugated antibodies against CD45, CD11b, and Ly6G for 45 min at 4°C and analyzed using a BD FACSVerse™ (gating strategy in Figure S1). Quantification was done with FlowJo V10 software.

### 2.4 Histology and microscopic immunofluorescence stainings

Cryo‐conserved lung sections were used for H&E (hematoxylin/eosin) staining or immunofluorescence analysis. For immunofluorescence, cells were fixed with 4% PFA in PBS, followed by drying, rehydration, and blocking with PBS containing 5% species‐specific serum and 1% BSA. Slides were incubated with primary antibodies (Ly6G, MPO, CD68) at 4°C overnight. After washing, Alexa‐fluor‐conjugated secondary antibodies and DAPI were added and incubated for 1 h. Vectashield® mounting medium was applied, and samples were analyzed using a Leica DMi8 microscope. Quantification involved analyzing 10 random images per slide for mean fluorescence intensity using ImageJ.

### 2.5 RT‐qPCR‐based mRNA expression analysis

mRNA was extracted from homogenized frozen lung tissues using a TRIzol™‐based protocol followed by RT‐qPCR using First Strand cDNA‐Synthesis‐Kit (ThermoFisher) and ORA™ SEE qPCR Green ROX‐H Mix. For primer pairs, see Supplementary Methods.

Relative mRNA levels were calculated using the ΔΔ^Ct^ method with *Rplp0* as housekeeping gene.

### 2.6 Analysis of BALF protein and Ccl2 levels

BALF protein content was analyzed using the bicinchoninic‐acid protein assay (ThermoFisher) and Ccl2 levels were measured with the Ccl2 DuoSet‐ELISA kit (R&D) using an EnSpire Multimode Plate Reader (PerkinElmer).

### 2.7 Statistical analysis

Statistical analyses were performed using GraphPad Prism Version 9 software. After testing for normal distribution (using Shapiro–Wilk testing), data were analyzed either by One‐way ANOVA or Kruskal‐Wallis test with Dunnett's, Dunn's or Tukey's post‐hoc test to adjust for multiple comparisons as appropriate. Data are presented as means ± SD. Data distributions with *p* < .05 were considered statistically significant.

## 3 RESULTS

### 3.1 MIF and MDL1 co‐inhalation synergistically enhances pulmonary recruitment of neutrophils and monocytic cells in vivo

**FIGURE 1:**
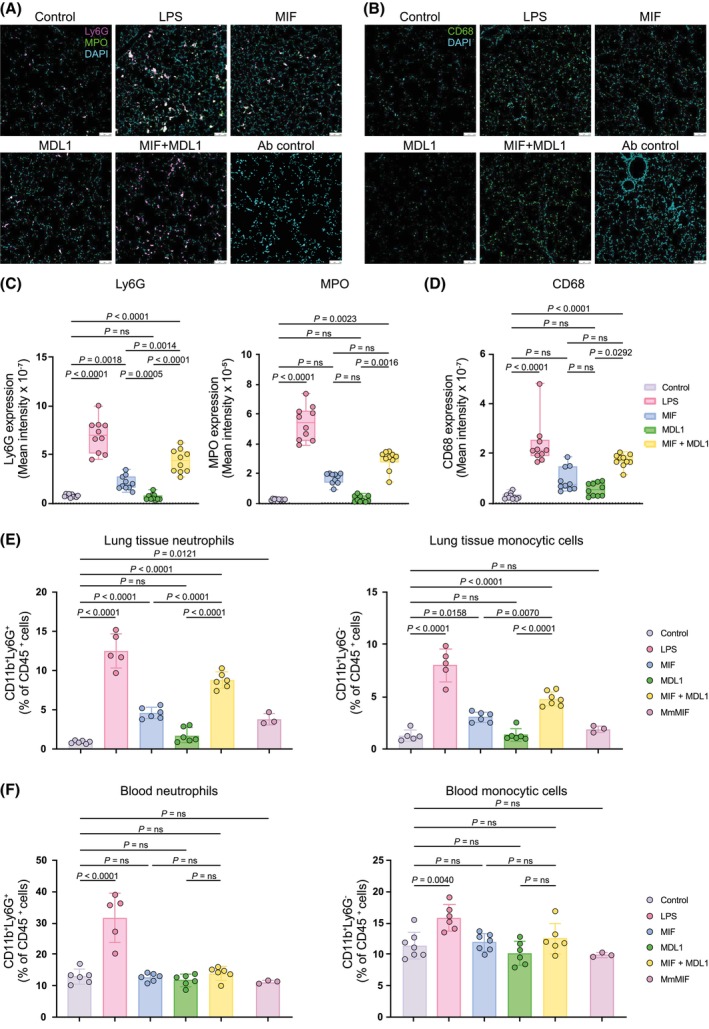
MDL1 amplifies MIF‐driven pulmonary infiltration of neutrophils and monocytic cells in an in vivo mouse model of acute lung injury (ALI). MDL1 amplified pulmonary leukocyte infiltration when co‐administered with human MIF, but not when applied alone. MIF, MDL1 or a combination of MIF and MDL1 (1:1) were applied to C57BL/6 mice via inhalation. After a 12 h incubation period, mice were sacrificed and pulmonary (lung tissue) neutrophil and monocytic cell levels were analyzed (A–D). Saline inhalation was used as negative control, LPS as positive control. To rule out species‐specific alterations, treatment with mouse MIF (MmMIF) was applied for comparison (E). (A) Representative fluorescent microscopy images as visualized by anti‐Ly6G (pink) and anti‐MPO (green) co‐staining. Cell nuclei were counterstained with DAPI (blue). Samples stained with secondary antibodies alone served as controls. Scale bar for all images is 100 μm (*n* = 10). (C) Quantification of the Ly6G^+^ and MPO^+^ mean fluorescence intensity according to (A); (data points from 10 randomly chosen images per mouse per experimental group). (B and D) Same as (A and C) except that infiltrated lung monocytic cells were visualized with anti‐CD68 antibody (green) and quantified. (E) Quantification of infiltrated cells by flow cytometry. Shown are neutrophils (left panel; CD45^+^CD11b^+^Ly6G^+^) and monocytic cells (right; CD45^+^CD11b^+^Ly6G^−^) as percentage of CD45^+^ cells. (F) Corresponding flow cytometry from blood showing circulating neutrophils (left panel; CD45^+^CD11b^+^Ly6G^+^) and monocytic cells (right; CD45^+^CD11b^+^Ly6G^−^). Values are shown as means ± SD with individual data points representing independent mice (*n* = 3–7). Statistical differences were analyzed using one‐way ANOVA with Tukey's post‐hoc test for E and F, with Dunnett's post‐hoc test for C (Ly6G) and Kruskal‐Wallis test with Dunn's post‐hoc test for C (MPO) and D. Statistical significance is indicated by *p* values. ns, not significant.

To explore the interplay between MIF and MDL1 on immune‐cell recruitment, cytokine expression, and lung injury, we adapted a mouse ALI model using inhalation of human MIF and MDL1 proteins, alone and combined. Pulmonary neutrophil and monocytic cell recruitment was assessed at 12 h post‐inhalation via immunofluorescence microscopy and flow cytometry (Figure 1). Treatment with LPS led to increased numbers of circulating and pulmonary neutrophils and monocytic cells, confirming induction of acute inflammation in this model (Figure 1). MIF significantly recruited neutrophils and monocytic cells into the lungs, although to a lesser extent than LPS and without affecting blood leukocyte numbers (Figure 1), suggesting localized pulmonary effects. Mouse MIF produced similar results (Figure 1E,F). Although MDL1 was previously found to promote neutrophil chemotaxis in vitro,^2^ MDL1 inhalation alone did not trigger significant immune‐cell recruitment in vivo. However, combined MIF and MDL1 inhalation enhanced pulmonary recruitment of both neutrophils and monocytic cells compared to individual treatments (Figure 1A–E), indicating synergistic activity.

### 3.2 Impact of MIF and MDL1 on pro‐inflammatory cytokine levels and parameters of lung injury

**FIGURE 2:**
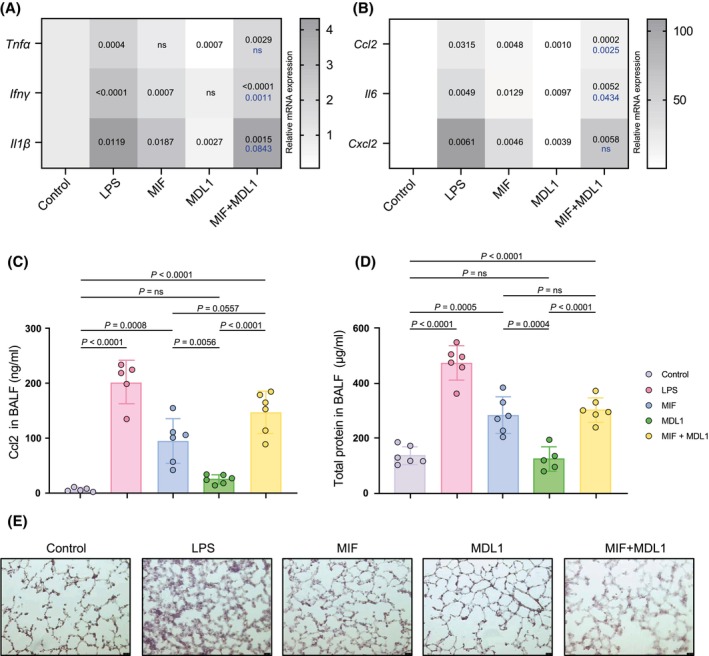
MDL1 amplifies MIF‐driven pro‐inflammatory cytokine levels in an in vivo mouse model of acute lung injury (ALI). (A and B) Heatmaps displaying (significant) changes in the mRNA expression of pro‐inflammatory cytokine genes using qRT‐PCR in C57BL/6 mouse lung tissue after nebulization with either human MIF, MDL 1 or the combination of MIF and MDL1 following an incubation period of 12 h. Saline inhalation is shown as negative control, inhalation of LPS served as positive control. Relative mRNA expression is shown relative to untreated controls with *Rplp0* used as housekeeping gene. Shown is the fold change expression of (A) *Tnf⍺*; *Ifnɣ*; *Il1β* and (B) *Ccl2*; *Il6*; *Cxcl2/Mip2* (*n* = 5–7, except *Tnf⍺* / MIF experimental group *n* = 1). Statistical significance is indicated by *p* values (black *p* values indicate significance in comparison to control group, blue *p* values indicate significance between MIF and combination of MIF and MDL1 experimental groups). ns, not significant. (C) Upregulation of CCL2 as verified by ELISA from bronchoalveolar fluid (BALF). (D) Total protein count in BALF served as a surrogate parameter for lung injury and was measured photometrically using a BCA assay (*n* = 5–6). (E) Representative images of H&E‐stained mice lungs after stimulation as indicated. Scale bar for all images: 25 μm. Values are shown as means ± SD with individual datapoints representing independent mice. Statistical analysis was performed using one‐way ANOVA with Tukey's post‐hoc test for A (*Tnf⍺ and Ifnɣ*) and Dunnett's post‐hoc test for A (*Il1β*), B (*Ccl2*, *Il6* and *Cxcl2/Mip2*), C and D. Statistical significance is indicated by *p* values. ns, not significant.

In addition to immune‐cell infiltration, pulmonary inflammation is characterized by upregulation of inflammatory mediators, including cytokines and chemokines. We assessed pro‐inflammatory gene expression in lung tissue using RT‐qPCR for *Tnfα, Ifnγ, Ccl2, Il1β, Il6*, and *Cxcl2/Mip2*. Inhalation of LPS upregulated the expression of all pro‐inflammatory cytokine mRNAs, while treatment with MIF elevated most of these, except for *Tnfα*, and to a lesser extent than LPS (Figure 2A,B). Again, MDL1 alone did not alter gene expression, but notably combined administration of MIF and MDL1 significantly amplified *Ifnγ, Ccl2*, and *Il6* gene expression beyond the levels induced by MIF alone. We next used ELISA to analyze BALF protein levels of Ccl2, a key monocyte chemokine also implicated in neutrophil recruitment, as an additional inflammation marker. Both LPS and MIF significantly increased Ccl2, with a close to significant (*p* = .0557) synergistic effect observed when MIF and MDL1 were combined (Figure 2C). Total BALF protein content is an important surrogate marker for lung injury, reflecting both passive capillary leak and active secretion of inflammatory mediators. BALF protein levels were significantly elevated following LPS and MIF exposure. Interestingly, the combination of MIF and MDL1 did not result in a significant increase in BALF protein compared to MIF alone (Figure 2D).

Lastly, histological analysis using H&E‐stained lung sections confirmed the extent of lung injury. Control mice exhibited thin alveolar walls with minimal cellular content within the alveoli. By contrast, LPS‐treated mice showed marked edematous thickening of alveolar walls and pronounced hypercellularity. Challenge with MIF induced similar changes, although less severe, while MDL1 alone did not cause notable morphological alterations. The combination of MIF and MDL1 led to changes similar to those seen with MIF alone (Figure 2E).

## 4 DISCUSSION

Here, we provide the first in vivo evidence of a synergistic interaction between the human cytokine/chemokine MIF and its *A. thaliana*‐derived ortholog MDL1 on pulmonary leukocyte trafficking and expression of pro‐inflammatory genes in an ALI/ARDS mouse model.

When considering prior biochemical evidence,^2^ three mechanisms may explain this synergy: (i) hetero‐oligomeric MIF/MDL1 complexes amplify leukocyte recruitment via CXCR2/4 with ensuing effects on the local inflammatory response and tissue damage; (ii) administered MIF enhances the pro‐inflammatory capacity of endogenous MIF, and this effect is amplified by MDL1 through complex formation or cooperative signaling; (iii) as the MIF/CD74 axis has been implicated in protective signaling in lung‐resident type II alveolar epithelial cells,^10^ it is conceivable that inflammatory CXCR2/4 pathways induced by MIF/MDL1 complexes may outweigh protective MIF/CD74 mechanisms. However, other studies have associated CD74 with increased ARDS severity, highlighting the complexity of MIF/CD74 signaling in lung injury.^11^, ^12^

Although our previous work showed that MDL1 can activate chemotaxis of primary human neutrophils in vitro, MDL1 inhalation alone showed no significant effects in our in vivo‐model. Species differences are unlikely, given the 90% sequence identity between mouse and human MIF. Instead, we assume endogenous pulmonary MIF levels were insufficient for efficient complex formation when MDL1 was inhaled alone. This hypothesis thus calls for future studies to explore pre‐existing lung inflammation/injury models with elevated MIF levels. Similarly, while our current work focused on local pulmonary effects of MIF/MDL1 in ALI, we haven't assessed systemic inflammatory parameters or performed leukocyte sub‐phenotyping, aspects to be addressed in future studies.

So far, studies on plant/mammalian MIF interactions have focused on *A. thaliana* MDLs, but conservation of MDLs across edible plants like *S. lycopersicum* (tomato) suggests a broader scope for future research. The herein demonstrated in vivo relevance of MDL/MIF interactions aligns with similar findings for parasite‐derived MIF orthologs, which have been identified as tractable pharmacological targets.^13^ While therapeutic strategies targeting MIF have focused on small molecules, peptides, and antibodies, modulating MIF‐MDL interactions could offer another potential strategy to mitigate certain MIF‐mediated diseases.^14^

In summary, our data demonstrate a pathologic effect of cross‐kingdom MIF/MDL interactions in an in vivo mouse model of ALI/ARDS, calling for future studies to further explore the translational relevance of such interactions in other MIF‐driven diseases.

## AUTHOR CONTRIBUTIONS

Lukas Spiller, Elias Lolis, Ralph Panstruga, Adrian Hoffmann, and Jürgen Bernhagen conceived and designed the study. Lukas Spiller, Lin Zhang, and Adrian Hoffmann performed research and analyzed data. Lukas Spiller, Lin Zhang, Patrick Scheiermann, Thierry Calandra, Elias Lolis, Ralph Panstruga, Adrian Hoffmann, and Jürgen Bernhagen contributed to the interpretation of the data. Simona Gerra contributed critical materials. The first draft of the manuscript was written by Adrian Hoffmann and Lukas Spiller with help from Jürgen Bernhagen. All authors critically reviewed, revised, and commented on the manuscript drafts and approved the final draft. Jürgen Bernhagen, Elias Lolis, Ralph Panstruga, and Adrian Hoffmann provided funding for the study.

## FUNDING INFORMATION

This work was supported by Deutsche Forschungsgemeinschaft (DFG) grants BE 1977/10‐1, BE 1977/17‐1, SFB1123‐A3, SFB1123‐A2, and by DFG under Germany's Excellence Strategy within the framework of the Munich Cluster for Systems Neurology (EXC 2145 SyNergy—ID 390857198) to J.B. A.H. was supported by a Metiphys scholarship of LMU Munich, funding by the Knowledge Transfer Fund (KTF) of the LMU Munich‐DFG excellence (LMUexc) program, and by the Friedrich‐Baur‐Foundation e.V. and associated foundations at LMU University Hospital. R.P. acknowledges funding from DFG grant PA 861/15‐1 and PA 861/24‐1, and C.S. from DFG grant STO 1099/8–1. E.L. received funding from Open Philanthropy. T.C. acknowledges support from the LMU Center for Advanced Sciences (CAS) program for visiting professors, and L.Z. was supported by a fellowship from the China Scholarship Council (CSC) program.

## DISCLOSURES

J.B. is the inventor on patent applications related to anti‐MIF strategies. All other authors declare no competing interests.

## ETHICS APPROVAL AND CONSENT TO PARTICIPATE

All mouse experiments were approved by the Animal Care and Use Committee of the local authorities (Regierung von Oberbayern, ROB; Aktenzeichen Az = ROB‐ 55.2Vet2532.Vet_02–18–40) and were performed in accordance with the animal welfare officer of the Center for Stroke and Dementia Research (CSD).

## Supporting information


Data S1.

## Data Availability

All data and materials as well as software application information are available in the manuscript, the supplementary information, or are available from the corresponding authors upon reasonable request.
