# Supplementary material for: In vivo synergistic enhancement of MIF‐mediated inflammation in acute lung injury by the plant ortholog Arabidopsis MDL1
Source: FASEB J. 2025 Mar 26;39(6):e70489. doi: 10.1096/fj.202403301R (PMC11937861; doi:10.1096/fj.202403301R)
Supplement: Supplementary file 1 — Data S1. [file FSB2-39-e70489-s001.pdf]

In vivo Synergistic Enhancement of MIF-mediated Inflammation in Acute Lung Injury by the Plant Ortholog Arabidopsis MDL1

Lukas Spiller[1,2,#], Lin Zhang[1], Simona Gerra[1], Christian Stoppe[3,4], Patrick Scheiermann[5], Thierry Calandra[1,6,7], Elias Lolis[2], Ralph Panstruga[8], Jürgen Bernhagen[1,9*], Adrian Hoffmann[1,5,9*]

[1] Division of Vascular Biology, Institute for Stroke and Dementia Research (ISD), LMU University Hospital, Ludwig-Maximilians-Universität (LMU) Munich, Munich, Germany
[2] Department of Pharmacology, School of Medicine, Yale University; New Haven, CT, USA Germany
[3] Department of Cardiac Anaesthesiology and Intensive Care Medicine, Charité, Berlin, Germany
[4] Department of Anaesthesiology, Intensive Care, Emergency and Pain Medicine, University Hospital Würzburg, Würzburg, Germany
[5] Department of Anaesthesiology, LMU University Hospital, Ludwig-Maximilians-Universität (LMU) Munich, Munich, Germany
[6] Service of Immunology and Allergy, Department of Medicine and Department of Laboratory Medicine and Pathology, Center for Human Immunology, Lausanne University Hospital, University of Lausanne, Lausanne, Switzerland
[7] Center for Advanced Studies, Ludwig-Maximilians-Universität (LMU) Munich, Munich, Germany
[8] Unit of Plant Molecular Cell Biology, Institute for Biology I, RWTH Aachen University, Aachen, Germany
[9] German Centre of Cardiovascular Research (DZHK), partner site Munich Heart Alliance, Munich, Germany.

*co-corresponding last authors
#current address:
Boehringer Ingelheim Pharma GmbH & Co. KG, Global Clinical Development & Operations
Binger Str. 173 | 55216 Ingelheim am Rhein, Germany

Correspondence:
Adrian Hoffmann, MD
Department of Anaesthesiology
LMU University Hospital
Ludwig-Maximilians-Universität (LMU) Munich
Marchioninistraße 15, 81377 Munich, Germany
Tel.: 0049-89 4400 - 16 - 81177
E-Mail: adrian.hoffmann@med.uni-muenchen.de

Professor Jürgen Bernhagen, PhD
Chair of Vascular Biology
Institute for Stroke and Dementia Research (ISD)
LMU University Hospital (LMU Klinikum)
Ludwig-Maximilians-Universität (LMU) München
Feodor-Lynen-Straße 17, 81377 Munich, Germany
Tel.: 0049-89 4400 - 46151
E-Mail: juergen.bernhagen@med.uni-muenchen.de

Supplementary Files

Supplementary Methods

- Detailed protocol protein expression
- Primer information qPCR

Supplementary Figures

- Supplementary Figure 1

Supplementary Methods

Detailed protocol protein expression

Recombinant human MIF and *A. thaliana* MDL1 proteins were produced using a bacterial overexpression system essentially as previously described (1-3). Briefly, *MIF* and *MDL1* were cloned into a pET21a vector and expressed in *E. coli* Rosetta (DE3) as His-tagged fusion construct. Induced fusion proteins MIF-6×His and MDL1-6×His were purified by fast protein liquid chromatography (FPLC; ÄKTA Pure, GE Healthcare/Cytiva, Germany) using a Nickel-loaded ion metal-affinity chromatography (IMAC) HisTrap column (GE Healthcare/Cytiva) followed by a Superdex 75 10/300 GL (GE Healthcare/Cytiva) size-exclusion chromatography (SEC) column. Purity was confirmed by SDS-PAGE and Western blotting, and endotoxin contamination ruled out by Pierce LAL Chromogenic Endotoxin Quantitation Kit (ThermoFisher Scientific, Germany). Recombinant mouse MIF was expressed and purified as described previously (2, 4).

Specific mouse primers used for RT-qPCR

*Tnfα*_forward (fwd) GGTGCCTATGTCTCAGCCTCTT,
*Tnfα*_reverse (rev) GCCATAGAACTGATGAGAGGGAG,
*Ifnγ*_fwd CAGCAACAGCAAGGCGAAAAAGG,
*Ifnγ*_rev TTTCCGCTTCCTGAGGCTGGAT
*Ccl2*_fwd GCTACAAGAGGATCACCAGCAG,
*Ccl2*_rev GTCTGGACCCATTCCTTCTTGG,
*Il1β*_fwd TGGACCTTCCAGGATGAGGACA,
*Il1β*_rev GTTCATCTCGGAGCCTGTAGTG,
*Il6*_fwd TACCACTTCACAAGTCGGAGGC,
*Il6*_rev CTGCAAGTGCATCATCGTTGTTC,
*Cxcl2/Mip2*_fwd CATCCAGAGCTTGAGTGTGACG,
*Cxcl2/Mip2*_rev GGCTTCAGGGTCAAGGCAAACT,
*Rplp0*_fwd GCTTCGTGTTCACCAAGGAGGA,
*Rplp0*_rev GTCCTAGACCAGTGTTCTGAGC.

<u>Supplementary Figures</u>

Supplementary Figure 1

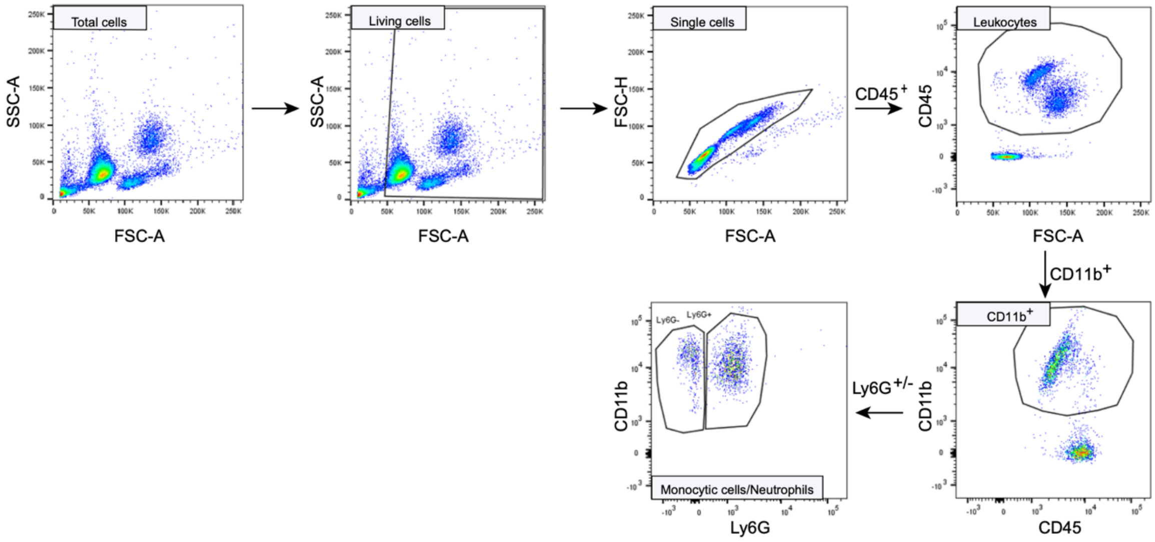

Supp. Figure 1. **Flow cytometry gating strategy.** Gating strategy for blood and lung neutrophils and monocytic cells. Visualization of a representative flow cytometry gating consisting of exclusion of debris, dead cells and doublets following selection of neutrophils ($CD45^{+}CD11b^{+}Ly6G^{+}$) and monocytic cells ($CD45^{+}CD11b^{+}Ly6G^{-}$).

References cited in Supplementary file.

1. Spiller, L., Manjula, R., Leissing, F., Basquin, J., Bourilhon, P., Sinitski, D., Brandhofer, M., Levecque, S., Gerra, S., Sabelleck, B., Zhang, L., Feederle, R., Flatley, A., Hoffmann, A., Panstruga, R., Bernhagen, J., and Lolis, E. (2023) Plant MDL proteins synergize with the cytokine MIF at CXCR2 and CXCR4 receptors in human cells. *Sci Signal* **16**, eadg2621
2. Bernhagen, J., Mitchell, R. A., Calandra, T., Voelter, W., Cerami, A., and Bucala, R. (1994) Purification, bioactivity, and secondary structure analysis of mouse and human macrophage migration inhibitory factor (MIF). *Biochemistry* **33**, 14144-14155
3. Sinitski, D., Gruner, K., Bernhagen, J., and Panstruga, R. (2020) Studying Plant MIF/D-DT-Like Genes and Proteins (MDLs). *Methods Mol Biol* **2080**, 249-261
4. Bernhagen, J., Calandra, T., Mitchell, R. A., Martin, S. B., Tracey, K. J., Voelter, W., Manogue, K. R., Cerami, A., and Bucala, R. (1993) MIF is a pituitary-derived cytokine that potentiates lethal endotoxaemia. *Nature* **365**, 756-759
